# Deep cytogenetics analysis reveals meiotic recombination depletion in species of *Senecio* (Asteraceae)

**DOI:** 10.1186/1999-3110-54-20

**Published:** 2013-08-27

**Authors:** Mariana G Lopez, Cecilia C Xifreda, Lidia Poggio, Arturo F Wulff

**Affiliations:** 1 CONICET, Laboratorio de Citogenética y Evolución (LACyE), Departamento de Ecología, Genética y Evolución, Facultad de Ciencias Exactas y Naturales, Universidad de Buenos Aires, Ciudad Universitaria, Pabellón 2, 4° piso, Int. Güiraldes 2620, C1428EHA Buenos Aires, Argentina; 2 CIC-PBA, Laboratorio de Etnobotánica y Botánica Aplicada (LEBA), Facultad de Ciencias Naturales y Museo, Universidad Nacional de La Plata, calle 64 N° 3, 1900, La Plata, Argentina; 3 Instituto de Biotecnología, Instituto Nacional de Tecnología Agropecuaria (INTA), Dr. N. Repetto y Los Reseros s/n, 1686 Hurlingham, Argentina; 4 Laboratorio de Citogenética y Evolución (LACyE), Departamento de Ecología, Genética y Evolución, Facultad de Ciencias Exactas y Naturales, Universidad de Buenos Aires, Ciudad Universitaria, Pab. II, 4° piso. Lab 61, Intendente Güiraldes 2160, (C1428EHA) Buenos Aires, Argentina

**Keywords:** Chiasmata, Chromosome number, Cytogenetics, Meiotic analysis, Meiotic recombination, *Senecio*

## Abstract

**Background:**

*Senecio* is the largest genus in the Asteraceae family growing in all environments around the world. It displays taxonomic and systematical difficulties. Cytogenetic knowledge of this genus is ancient, scarce and mainly restricted to chromosome number records.

**Results:**

In this study we analyzed chromosome number, meiotic configuration, bivalent morphology, meiotic behavior and pollen grain stainability on 100 accessions of 27 different polyploid *Senecio* L. sect *Senecio* entities. Median, standard deviation and mode were calculated for number and position of chiasmata and meiotic recombination was statistically evaluated. Although high frequency of multivalents and associated meiotic irregularities are expected in high polyploids, bivalents predominance and, consequently, regular meiosis were observed, with normal sporogenesis and high pollen grain stainability.

**Conclusion:**

Depletion in the total chiasmata was significant only in some species but the terminal position was preferential in all the entities analyzed, indicating significant reduction in recombination. The regular meiosis observed suggest that intra and intergenomic reorganization process occur quickly and efficiently in this genus. Mechanisms of diploidization, common to all polyploids, are reinforced by the strong reduction in crossing-over rushing polyploids stabilization.

**Electronic supplementary material:**

The online version of this article (doi:10.1186/1999-3110-54-20) contains supplementary material, which is available to authorized users.

## Background

*Senecio* L. is a cosmopolitan genus described by Linné in 1753. It grows in all the regions with the exception of South Pacific Islands and Antarctica, although shows the highest specific richness in the mountain areas (Cabrera et al., 1999). The number of species belonging to *Senecio* is controversial, ranging from 1000 to up 3000, mainly because the extremely difficult taxonomy and systematics. Nevertheless this debate, different authors agree that it is the largest genus of the Asteraceae family (Bremer 1994; Cabrera et al., 1999; Nordenstam, 2007; Pelser et al., 2007). Medicinal, ornamental, noxious plants and weeds fit into this group giving it, in addition to the ecological value, an economical interest. At the cytogenetic level the modal chromosome numbers in *Senecio* are 2n = 40 and 2n = 80 (Bolkhovskikh et al., 1974). Polyploidy is one of the most notorious features in the genus (Lawrence, 1980; 1985) and x = 5, the accepted basic chromosome number (López et al., 2008a). Even when in the last years some works were published, mostly about Argentine species (López et al., 2002; 2005; 2008a; 2008b) cytogenetic knowledge of *Senecio* in the world is still scarce and ancient (see López, Citogenetics, Evolutive and Biosistematical Studies in Senecio sect. Senecio serie Corymbosi, from Argentina. 2008. Unpublished PhD Thesis University of Buenos Aires. Argentina for complete revision) and mainly restricted to chromosome number records. It has been suggested that deeper cytogenetic analyses would be very useful in order to understand the evolution and clarify the taxonomic complexity in this diverse taxon (López et al., 2008a; 2008b).

Polyploidy has received special interest during the last decade, assigning it the main role in relation to the evolution of the angiosperms. It is considered an important speciogenic force and the major source of genetic variability in plants (Heslop-Harrison Pat and Schwarzacher 2011). Otherwise the old thoughts emphasized variability in the combination of genomes occurring in polyploids (Stebbins, 1980; Grant, 1989), more recent approaches highlight the recurrent formation of polyploids (Ashton and Abott 1992), the huge genomic reorganization caused by the interaction between genomes and the epigenetic modification as central resources of variability associated to polyploidy (Soltis and Soltis, 1999; Wendel 2000; Soltis et al., 2003; Kovarik et al., 2005; Tate et al., 2005; Hegarty et al., 2006; Chen et al. 2007).

A lot of knowledge about *Senecio* has been generated from molecular data. On the other hand, cytogenetic behavior in polyploids and the consequences of the chromosome genomic rearrangement are less understood. In this sense, previous studies revealed chiasmata excess in taxa from this genus and suggested a chromosome exchange preferentially placed in terminal position (López et al., 2005). Since both events are determinant in the chromosome recombination, their estimation result extremely interesting because could explain the sterility avoidance caused by meiotic alterations, and therefore the maintenance and success of polyploidy species in the nature.

Since *Senecio* is a genus plenty of polyploid species and notably successful worldwide, our main interest is to present a detailed cytogenetic characterization of several of its species, with special emphasis in the meiotic features, with the objective of understand the task of chromosomes in their success and permanence.

## Methods

### Plant material

Plant materials, including 27 entities, were collected between 2001 and 2006 in different provinces of Argentina (Appendix 1). Voucher specimens were identified, labeled and deposited at the herbaria SI or BAFC. Young capitula were fixed *in situ* in ethanol-chloroform-glacial acetic acid (6:3:1) for at least 24 hs, transferred to ethanol 70% (v/v) and stored at 4°C until used.

### Meiotic studies

Preparations were obtained squashing immature anthers in a drop of a 2% propionic acidhaematoxylin solution and using ferric acid as mordant (Núñez 1968). Cytogenetic analyses were performed taking into account a minimum of 50 pollen mother cells (PMCs) per species at different meiotic stages. Slides were observed and photographed using an optic photomicroscope Leica BMLD and a Leica DFC 350 FX digital camera. This study included determination of chromosome numbers, meiotic configurations, bivalents morphology and meiotic behavior. Median, standard deviation and mode were calculated for the number and the position of chiasmata. Pollen grain stainability was studied with Alexander’s differential staining method (Alexander, 1969).

In order to evaluate statistically the meiotic recombination, we apply a sample population proportion test (Daniel, 1999). The statistic “z” used in this test was calculated with the following formula:

$$z=\frac{\hat{p}-p}{\sqrt{\frac{\hat{p}\hat{q}}{n}}}\;\begin{array}{c} \hat{p}=0,5\;\\ p=1‒\hat{p}=0,5 \end{array}$$

We tested two hypotheses, taking into consideration that the number and the position of chiasmata occurs randomly (i.e. p = 0.5), as is expected in the meiotic process. For the first hypothesis, z1 was defined as the proportion of open bivalents and in the second one, z2, as the proportion of interstitial chiasmata. In order to minimize the differences in chiasmata occurrence during meiosis, open bivalents and chiasmata position were determined mainly in diakinesis and also in prometaphase I, in cells where those items could be undoubtedly determined. Previous published chromosome number reports were included in this report in order to enlarging the discussion of this cytogenetic study and the meiotic recombination analysis.

## Results

### Chromosome numbers and meiotic configurations

**Figure 1:**
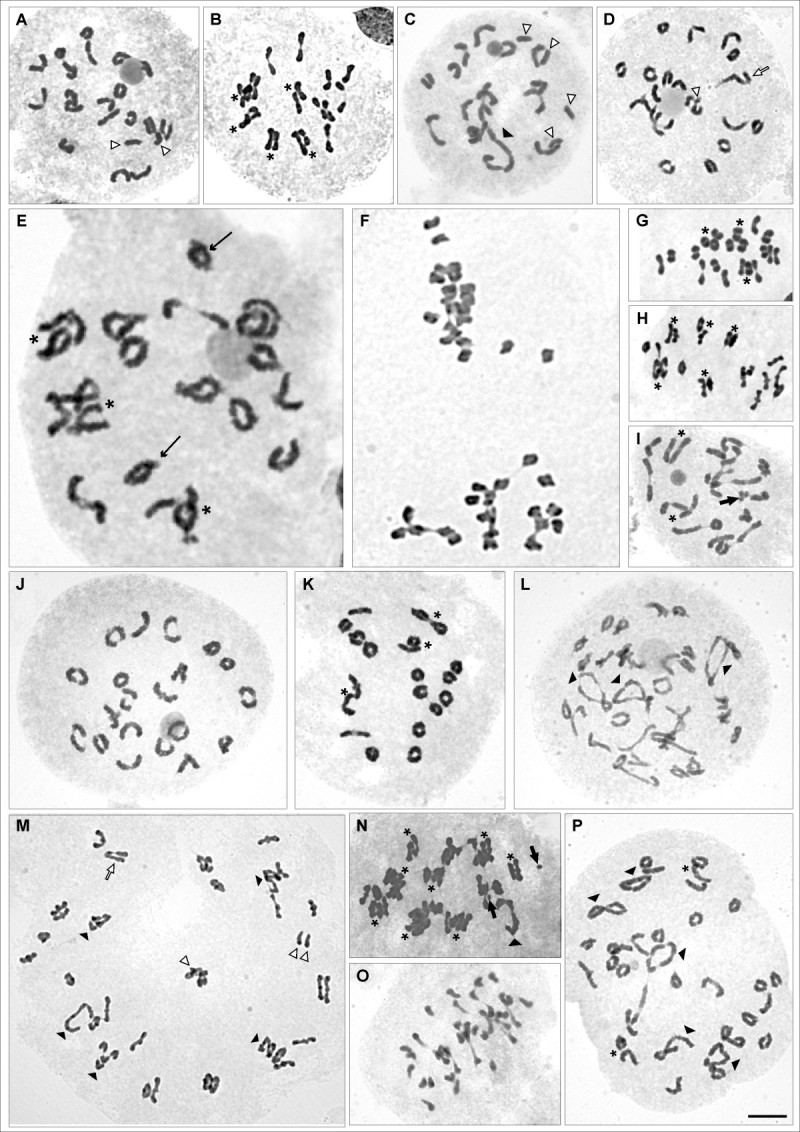
**Cytogenetic analysis of** ***Senecio*** **sect.** ***Senecio*** **taxa. A**: *S. eruciformis* var. *brachycephalus*, diakinesis with 19 II + 2 I. **B**: *S. eruciformis* var. *eruciformis*, metaphase I with 20 II. **C**: *S. grisebachii* var. *schizotus*, diakinesis with 16 II + 1IV + 4 I. **D**: *S. linariifolius* var. *subtomentosus*, diakinesis with 18 II + 1 III + 1 I. **E**: *S. glaber*, diakinesis with 20 II. **F**: *S. riojanus*, prometaphase II with 20 chromosomes in each pole. **G**: *S. ganganensis*, prometaphase I with 20 II. **H**: *S. goldsackii*, metaphase I with 20 II. **I**: *S. grisebachii* var. *leptotus*, diakinesis with 20 II + 3 B chromosomes (1 IIB + 1 IB). **J**: *S. brasiliensis* var. *tripartitus*, diakinesis with 20 II. **K**: *S. melanopotamicus*, diakinesis with 20 II. **L** *S. subulatus* var. *salsus*, diakinesis with 34 II + 3 IV. **M**: *S. viridis* var. *viridis*, prometaphase I with 27 II + 5 IV + 1 III + 3 I, displaying evident secondary association of bivalents. **N**: *S. microphyllus*, metaphase I with 38 II + 1 IV + 2 IB. **O**: *S. subulatus* var *erectus* cytotype 2n = 40, late metaphase I with 20 II. **P**: *S. subulatus* var. *erectus* cytotype 2n = 80, diakinesis with 30 II + 5 IV. Asterisks indicate secondary association of bivalents; white triangles indicate univalents (I); line arrows indicate heteromorphic bivalents; black triangles indicate quadrivalents (IV); tick white arrows indicate trivalents (III); thick black arrows indicate B chromosomes; c: close bivalents; qi: interstitial chiasmata. Bars = 10 μm.

Complete cytogenetic analysis is summarized in Table 1. Only two different sporophytic numbers (2n) were observed, 2n = 40 and 2n = 80. New records on chromosome number were presented for: *S*. *eruciformis* var. *brachycephalus* (Figure 1A), *S*. *eruciformis* var. *eruciformis* (Figure 1B), *S*. *grisebachii* var. *schizotus* (Figure 1C), *S*. *linariifolius* var. *subtomentosus* (Figure 1D), *S*. *glaber* (Figure 1E), *S*. *riojanus* (Figure 1F), *S*. *ganganensis* (Figure 1G), *S*. *goldsackii* (Figure 1H), *S*. *grisebachii* var. *leptotus* (Figure 1I); *S. brasiliensis* var. *tripartitus* (Figure 1J) and *S*. *melanopotamicus* (Figure 1K) with 2n = 40; *S*. *subulatus* var. *salsus* (Figure1L), *S*. *viridis* var. *viridis* (Figure 1M) and *S*. *microphyllus* (Figure 1N) with 2n = 80 and, *S*. *subulatus* var. *erectus* which exhibited cytotypes with both chromosome numbers (Figure 1O-P, Table 1). Polymorphism for B chromosomes was also observed in some entities (Table 1, Figure 1I, 1N). Although diverse meiotic figures were identified, from univalents (I) to hexavalents (VI) and one decavalent (X), bivalent (II) was the most frequent chromosome pairing (Table 1, Figure 1A-P). Interstitial chiasmata and close bivalents were also marked in the cells used to recombinant analysis (Figure 1A-P).

**Table 1:** **Meiotic analysis and pollen stainability of entities belonging to different Subseries of** ***Senecio*** **sect.** ***Senecio***

Species	2n	Meiotic configuration	Chiasmata/cell		Interstitial ch.		Pollen stainability
			$\bar{x}$ ± sd	M	$\bar{x}$ ± sd	M	
**Subserie Brasiliensis (Cabrera) Cabrera et S.E. Freire**							
*S. brasiliensis* var. tripartitus** (4, 28)	40	20 II (92.8%)	27.5 ± 2.4	30	4.2 ± 2.3	4	96.1 - 99.4%
		19 II + 2 I (3.6%)	(z_1_ = −1.04)		(z_2_ = 4.99)^++^		
		18 II + 4 I (3.6%)			(0–9)		
*S. eruciformis* var. brachycephalus** (2, 70)	40	20 II (87.1%)	24.9 ± 3.5	24	3.0 ± 2.2	2	98.4 - 99.6%
		19 II + 2 I (10%)	(z_1_ = −2.19)^++^		(z_2_ = 5.37)^++^		
		17 II + 1 IV + 2 I (1.45%)			(0–9)		
		18 II + 1 III + 1 I (1.45%)					
*S. eruciformis* var. eruciformis** (2, 60)	40	20 II (91.7%)	26.1 ± 2.8	29	2.6 ± 2.6	1	97.4 - 98.3%
		19 II + 2 I (8.3%)	(z_1_ = −1.63)		(z_2_ = 5.49)^++^		
					(0–12)		
S. ganganensis** (1, 45)	40	20 II (93.3%)	29.5 ± 2.0	30	3.3 ± 2.1	4	98.90%
		18 II + 4 I (4.5%)	(z_1_ = −0.11)		(z_2_ = 5.28)^++^		
		19 II + 2 I (2.2%)			(0–9)		
*S. gilliesianus* (4, 54)	40	20 II (94.45%)	31.7 ± 3.2	33	8.8 ± 2.1	10	92.1 - 98.8%
		19 II + 2 I (1.85%)	(z_1_ = 0.81)		(z_2_ = 3.52)^++^		
		18 II + 1 IV (1.85%)			(2–17)		
		16 II + 2 IV (1.85%)					
S. glaber** (1, 17)	40	20 II (100%)	32.0 ± 2.2	31	6.7 ± 4.2	3	83.40%
			(z_1_ = 0.89)		(z_2_ = 4.20)^++^		
					(1–14)		
S. goldsackii** (3, 79)	40	20 II (94.9%)	30.9 ± 2.4	31	9.2 ± 3.6	8	96.7 - 99.5%
		18 II + 1 IV (3.8%)	(z_1_ = 0.42)		(z_2_ = 3.40)^++^		
		19 II + 2 I (1.3%)			(2–19)		
S. melanopotamicus** (6, 76)	40 + 0-1B	20 II (98.7%)	33.5 ± 2.1	33	8.2 ± 3.5	10	93.7 - 99.6%
		18 II + 1 IV (1.3%)	(z_1_ = −1.56)		(z_2_ = 3.74)^++^		
					(0–16)		
S. microphyllus** (2, 10)	80 + 0-6B	40 II (60%)	47.2 ± 5.8	46	4.6 ± 3.3	2	98.5 - 99.5%
		38 II + 1 IV (30%)	(z_1_ = −3.64)^++^		(z_2_ = 4.86)^++^		
		36 II + 2 IV (10%)			(1–9)		
*S. pampeanus* (3, 24)	40 + 0-1B	20 II (75%)	27.0 ± 2.6	27	2.5 ± 2.1	1	98.3 - 98.7%
		19 II + 2 I (16.7%)	(z_1_ = −0.99)		(z_2_ = 5.55)^++^		
		17 II + 6 I (4.15%)			(0–7)		
		18 II + 1 IV (4.15%)					
*S. pinnatus* (4, 36)	80 + 0-10B	40 II (36.1%)	59.7 ± 3.6	60	10.4 ± 4.2	11	98.8 - 99.6%
		38 II + 1 IV (22.2%)	(z_1_ = −0.06)		(z_2_ = 6.62)^++^		
		36 II + 2 IV (19.4%)			(3–20)		
		32 II + 4 IV (8.3%)					
		34 II + 3 IV (5.5%)					
		38 II + 1 III + 1 I (5.5%)					
		32 II + 3 IV + 4 I (3%)					
S. ragonesei* (3, 87)	40	20 II (96.6%)	30.4 ± 1.6	30	4.9 ± 2.1	5	81.6 - 98.6%
		19 II + 2 I (3.4%)	(z_1_ = 0.23)		(z_2_ = 4.76)^++^		
					(1–10)		
S. riojanus ** (1, 1)	40	without data					99.40%
*S. rudbeckiifolius* (7, 98)	40 + 0-6B	20 II (85.8%)	28.8 ± 2.5	30	4.6 ± 2.4	4	98.2 -98.7%
		18 II + 1 IV (7.1%)	(z_1_ = −0.48)		(z_2_ = 4.88)^++^		
		16 II + 2 IV (3.1%)			(0–13)		
		19 II + 2 I (2%)					
		18 II + 4 I (1%)					
		15 II + 1 IV + 1 VI (1%)					
*S. subulatus* var. erectus** (1, 4) (6,43)	40	20 II (100%)	24.5 ± 3.0	27	1.2 ± 1.5	0	71.50%
			(z_1_ = −2.46)^++^		(z_2_ = 5.93)^++^		
					(0–3)		
	80 + 0-9B	38 II + 1 IV (34.9%)	54.4 ± 6.6	55	5.3 ± 3.7	3	90 - 95%
		40 II (27.8%)	(z_1_ = −1.57)		(z_2_ = 7.75)^++^		
		36 II + 2 IV (12%)			(1–14)		
		34 II + 3 IV (4.6%)					
		30 II + 5 IV (4.6%)					
		37 II + 1 IV + 2 I (2.3%)					
		39 II + 2 I (2.3%)					
		38 II + 4 I (2.3%)					
		35 II + 1 VI + 1 IV (2.3%)					
		35 II + 1 IV + 2 III (2.3%)					
		35 II + 2 IV + 2 I (2.3%)					
		32 II + 4 IV(2.3%)					
S. subulatus var. salsus** (1, 10)	80	34 II + 3 IV (50%)	62.0 ± 4.8	54	11.5 ± 3.6	16	98.50%
		36 II + 2 IV (30%)	(z_1_ = 0.76)		(z_2_ = 6.36)^++^		
		32 II + 4 IV (20%)			(7–16)		
*S. subulatus* var. subulatus* (4, 81) (3,16)	40 + 0-1B	20 II (95.1%)	21.1 ± 4.1	25	3.4 ± 2.8	1	90 - 99.3%
		19 II + 2 I (3.7%)	(z_1_ = −1.25)		(z_2_ = 5.23)^++^		
		18 II + 1 IV (1.2%)			(0–13)		
	80	38 II + 1 IV (25%)	55.9 ± 3.2	58	7.3 ± 4.5	5	90 - 99.4%
		34 II + 3 IV (25%)	(z_1_ = −1.01)		(z_2_ = 7.30)^++^		
		36 II + 2 IV (18.75%)			(1–20)		
		40 II (6.25%)					
		32 II + 4 IV (6.25%)					
		39 II + 2 I (6.25%)					
		37 II + 1 IV + 2 I (6.25%)					
		32 II + 3 IV + 4 I (6.25%)					
*S. uspallatensis* (13, 123)	80 + 0-5B	40 II (34.1%)	52.7 ± 5.8	52	7.6 ± 4.8	5	72.9 - 99.9%
		38 II + 1 IV (21.95%)	(z_1_ = −2.21)^++^		(z_2_ = 7.23)^++^		
		36 II + 2 IV (20.32%)			(0–21)		
		34 II + 3 IV (4.95%)					
		39 II + 2 I (3.25%)					
		37 II + 1 IV + 2 I (3.25%)					
		34 II + 2 IV + 1 III + 1 I (1.63%)					
		35 II + 2 IV + 2 I (1.63%)					
		32 II + 4 IV (1.63%)					
		38 II + 4 I (0.81%)					
		36 II + 1 IV + 4 I (0.81%)					
		33 II + 3 IV + 2 I (0.81%)					
		33 II + 2 IV + 2 III (0.81%)					
		33 II + 1 VI + 2 IV (0.81%)					
		32 II + 3 IV + 4 I (0.81%)					
		31 II + 1 X + 2 IV (0.81%)					
		31 II + 1VI + 2 IV + 1 III + 1 I (0.81%)					
		30 II + 5 IV (0.81%)					
**Serie Sandwithiani (Cabrera) Cabrera et S.E.Freire**							
*S. viridis* var. radiatus* (8, 143)	40 + 0-1B	20 II (94.4%)	31.4 ± 3.0	32	5.6 ± 3.0	6	94.1 - 99.7%
		18 II + 1 IV (3.5%)	(z_1_ = 0.68)		(z_2_ = 4.56)^++^		
		19 II + 2 I (2.1%)			(0–13)		
*S. viridis* var. viridis** (1, 19)	80 + 0-2B	34 II + 3 IV (21.1%)	58.7 ± 2.2	61	13.7 ± 3.9	12	98.90%
		32 II + 4 IV (21%)	(z_1_ = 0.08)		(z_2_ = 5.88)^++^		
		40 II (10.4%)			(8–21)		
		38 II + 1 IV (10.4%)					
		39 II + 2 I (5.3%)					
		36 II + 2 IV (5.3%)					
		36 II + 1 IV + 1 III + 1 I (5.3%)					
		30 II + 5 IV (5.3%)					
		30 II + 4 IV + 1 III + 1 I (5.3%)					
		28 II + 5 IV + 1 III + 1 I (5.3%)					
		24 II + 7 IV + 1 III + 1 I (5.3%)					
**Serie Simplices (Cabrera) Cabrera et S.E.Freire**							
*S. grisebachii* var. *grisebachii* (1, 9)	40	20 II (100%)	27.9 ± 2.6	28	6.0 ± 2.3	7	91.50%
			(z_1_ = −0.94)		(z_2_ = 4.43)^++^		
					(2–9)		
*S. grisebachii* var. leptotus** (5, 57)	40 + 0-2B	20 II (98.2%)	24.8 ± 3.1	21	3.7 ± 2.2	4	97.8 - 99.8%
		19 II + 2 I (1.8%)	(z_1_ = −2.31)^++^		(z_2_ = 5.15)^++^		
					(0–10)		
*S. grisebachii* var. schizotus** (3,37)	40	20 II (75.7%)	24.1 ± 2.6	24	4.2 ± 2.3	4	97.5 - 99.5%
		19 II + 2 I (10.8%)	(z_1_ = −2.36)^++^		(z_2_ = 5.01)^++^		
		18 II + 1 IV (8.1%)			(0–9)		
		18 II + 4 I (5.4%)					
*S. hieronymi* (5, 90)	40 + 0-7B	20 II (90%)	28.4 ± 2.9	29	3.8 ± 2.0	3	98.60%
		19 II + 2 I (6.7%)	(z_1_ = −0.64)		(z_2_ = 5.12) ^++^		
		18 II + 1 IV (3.3%)			(0–9)		
*S. linariifolius* var. subtomentosus** (1, 46)	40	20 II (87%)	28.0 ± 2.6	28	3.2 ± 2.3	5	without data
		19 II + 2 I (8.7%)	(z_1_ = −0.82)		(z_2_ = 5.32)^++^		
		18 II + 1 III + 1 I (2.15%)			(0–9)		
		18 II + 1 IV (2.15%)					
*S. octolepis* var. saltensis* (2, 41)	40	20 II (92.7%)	25.1 ± 3.0	24	4.8 ± 2.0	6	94.4 - 99.1%
		16 II + 2 IV (4.9%)	(z_1_ = −2.16)^++^		(z_2_ = 4.82)^++^		
		18 II + 1 IV (2.4%)			(1–10)		
**Serie Viscosi (Baker) Cabrera et S.E.Freire**							
S. crepidifolius* (1, 17)	40	20 II (88.2%)	25.8 ± 2.8	26	4.2 ± 2.2	2	without data
		19 II + 2 I (5.9%)	(z_1_ = −1.73)		(z_2_ = 4.98)^++^		
		18 II + 4 I (5.9%)			(1–9)		

In the first column. numbers in parentheses indicate the number of individuals and cells. respectively. analyzed in each taxon. Chromosome number (2n); meiotic configurations and corresponding percentage. Media ($\bar{x}$). standard deviation (sd) and mode (M) of total chiasmata per cell and interstitial chiasmata (interstitical ch.). range of instestitial chiasmata in parentheses. both statistics values z_1_ and z_2_ and percentage of pollen stainability are shown.

*chromosome record already published by the authors.

**new chromosome record.

^++^significant values (p < 0.05).

z_1_: proportion of open bivalents. z_2_: proportion of interstitial chiasmata.

### Meiotic behavior

**Figure 2:**
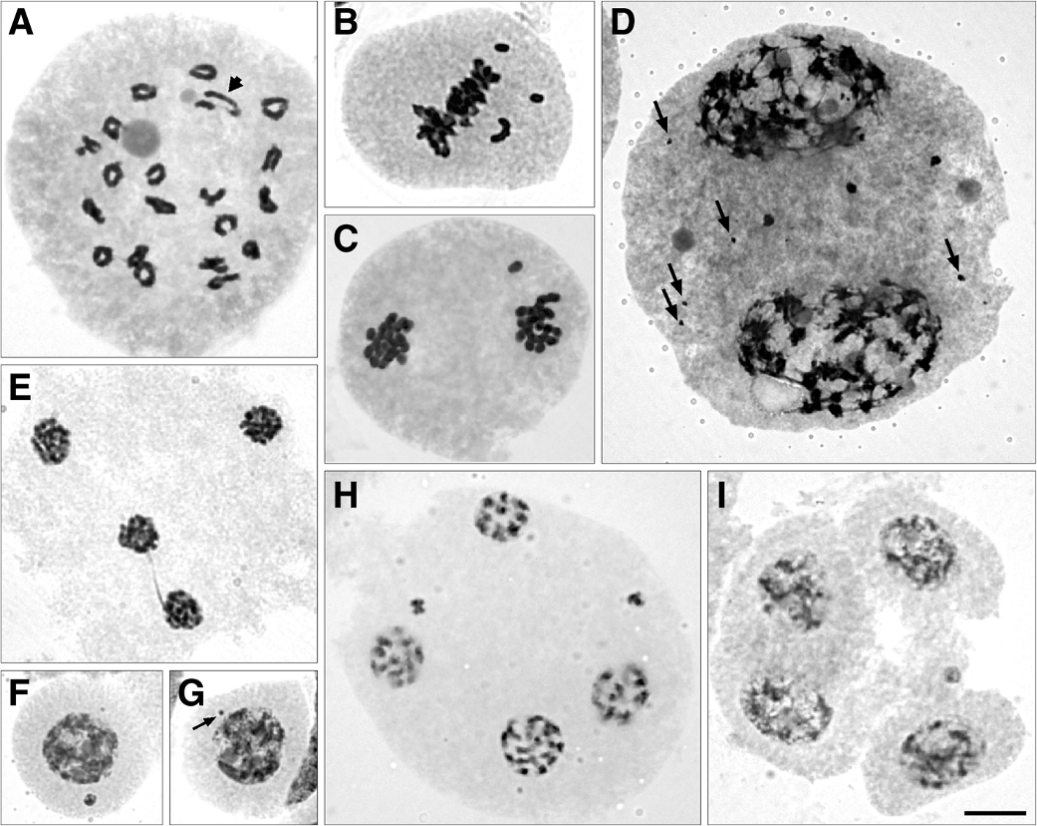
**Meiotic behavior of** ***Senecio*** **sect.** ***Senecio*** **taxa. A**: *S. melanopotamicus*, diakinesis with 20 II, arrow indicate chromosome breakage. **B**: *S. goldsackii,* metaphase I, 1 II and 2 I out of plate. **C**: *S. melanopotamicus*, telophase I, a chromosome not included in a pole. **D**: *S. microphyllus,* prophase II with A and B (arrows) chromosomes not included in cell poles. **E**: *S. goldsackii*, telophase II with a bridge. **F-G**: *S. subulatus* var. *erectus,* **F**: microsporocyte with one big micronucleus (A chromosome); **G**: microsporocyte with one small micronucleus (arrow, B chromosome). **H**: *S. ganganensis,* telophase II with micronuclei. **I**: *S. eruciformis* var. *brachycephalus*, tetrads with one micronucleus. Bars = 10 μm.

Meiotic behavior was carefully examined in all the new recorded entities except in *S*. *riojanus* because of the scarce material in adequate maturation condition. The majority of the individuals exhibited bivalents secondarily associated (Figure 1B, E, G-I, K, M-N, P). Heteromorphic bivalents (Figure 1E) were observed in all the taxa with 2n = 40, with exception of *S*. *ganganensis*, *S*. *linariifolius* var. *subtomentosus* and *S*. *subulatus* var. *salsus*. Completely regular meiosis was seen in *Senecio eruciformis* var. *brachycephalus*, *S*. *ganganensis*, *S*. *goldsackii*, *S*. *grisebachii* var. *schizotus*, *S*. *viridis* var. *viridis* and *S*. *subulatus* var. *erectus*. In some species, meiotic irregularities such as, chromosome breakage (Figure 2A); bivalents out of plate at metaphase I (Figure 2B); lagged chromosomes at anaphase I and II; chromosomes excluded to the nucleus at telophase I (Figure 2C) and prophase II (Figure 2D); bridges at Telophase II (Figure 2E); micronuclei at Telophase I, Prophase II, Telophase II (Figure 2H), tetrads (Figure 2I) and microsporocytes (Figure 2F, G) were observed, although infrequently. In species with B chromosomes, they were excluded from the nucleus and remain as micronuclei after Telophase. Meiotic behavior of the other entities included in this paper was described in previous publications (López et al., 2002; 2005; 2008b). Pollen stainability was higher than 70% in all the species.

### Recombination analysis

Values of median and mode of chiasmata per cell were similar in all the cases. The statistics z1 was significant (p < 0.05) only in 7 entities (Table 1) and negative in all of them, indicating that the number of open bivalents was higher than the expected by random.

Median and mode of interstitial chiasmata per cell were low for all species analyzed, ranging from 1.2 to 13.7 and from 0 to16 respectively, with high data dispersion. The statistics z2 was significant (p < 0.05) and positive in all cases, indicating a reduction in the interstitial chiasmata in relation with the expected by random, and as a consequence the predominance of chiasmata in terminal position.

## Discussion

Strong evidence supporting x = 5 as the basic chromosome number in the genus *Senecio* has been recently published (López et al., 2008a). Considering this statement, the species here studied displayed ploidy levels 8x and 16x, and, chromosome numbers 2n = 40 and 2n = 80 respectively, both of them in accordance with the modal numbers of the genus (Bolkhovskikh et al., 1974). It is noticeable the existence of two *S*. *subulatus* varieties presenting both chromosome numbers. These data represent the first reports of cytotypes for this genus in Argentina. This polymorphic species comprises three varieties coexisting sympatrically in nature, mostly in Mendoza province, but practically indistinguishable, except by minor differences in the capitula. Further analyses of the infraspecific taxa will contribute to solve the taxonomic and cytogenetic controversies but the existence of different ecological or environmental patterns could explain the presence of both cytotypes in the same place (Schönswetter et al., 2007; Suda et al., 2007).

Even when high frequency of multivalents and the meiotic irregularities associated are expected in elevated polyploids, bivalents predominance and consequently regular meiosis were observed in this analysis and in the previous ones conducted in *Senecio* (López et al., 2002; 2005; 2008a). The documented meiotic irregularities can be mainly consequence of the structural rearrangements occurring between genomes, such as translocations, paracentric inversions, deletions, and insertions among others, which have been recorded for other polyploids by several authors (Stebbins, 1980; Thompson and Lumaret, 1992; Soltis and Soltis, 1993; Comes and Abbott, 1999; López et al., 2002). Frequently, the lagged chromosomes and the micronuclei were associated with B chromosomes suggesting a particular behavior of them to remain excluded from the nucleus. The scarce meiotic irregularities had not, apparently effect over pollen grain integrity as revealing by the high stainaiblity values observed.

The abundance of bivalents linked to their outstanding secondary association revealed a diploidized behavior in these species and, this was in accordance with data previously published (Riley and Chapman 1958; López et al., 2002; 2008a). This particularity could be interpreted as reduction in crossing-over and consequently decrease in the number chiasmata per cell. Depletion in the total chiasmata was significant only in some species but the terminal position was preferential in all the *Senecio* entities analyzed. Both parameters indicate a significant reduction in recombination. In the first case, less chiasmata imply clearly less crossing-over in the second case, restriction to the terminal position limit the interchange to a small segment, keeping most of the chromosome length almost invariable.

Although these polyploids underwent a substantial reduction in genetic recombination, there was not a notorius loss of variability sentencing these species to disappearance. On one hand, chromosome orientation in metaphase I is a source of variability and it is not affected by polyploidy. Conversely, the increase in chromosome number enlarges the potential combinations. On the other hand, polyploids have exclusive resources to obtain variability including hybridization, recurrent formation, genomic reorganization and new expression patterns (Soltis and Soltis, 1993; 1999; Wendel, 2000; Soltis et al., 2003; Kovarik et al., 2005; Tate et al., 2005; Hegarty *et al*., 2006; Chen et al. 2007; López et al., 2008b). In addition, recombination restricted to chromosome terminal positions could favor the maintenance of adaptative gene combinations over generations.

The surprisingly regular meiosis observed would suggest that intra and intergenomic reorganization process occur quickly and efficiently in the genus *Senecio* modifying chromosome homology and probably favoring a diploidized behavior. B chromosomes have been linked with this particular chromosome behavior (Gupta, 1981), but this relationship is not evident in *Senecio*. Moreover, a different factor has been proposed in relation with it, the existence of Ph like genes (Moore, 1998; Sybenga, 1999; Al-Kaff et al., 2008). Although they have not been described yet in the genus *Senecio,* their effect on the promoting of diploidized behavior could not be discarded.

## Conclusion

Processes producing diploidized behavior avoid the sterility associated to high polyploids and maintain advantage adaptative allele combinations, ensuring species success, stabilization and establishment in nature (Stebbins, 1980; Thompson and Lumaret, 1992; Comes and Abbott 1999; Soltis and Soltis, 1993,1999). Especially, in the genus *Senecio,* the mechanisms of diploidization are accelerated by a strong reduction in crossing over, process evidenced in the present work.

## Appendix 1

List of *Senecio* specimens analysed, collection data, voucher number and herbaria where they are deposited in parentheses. All the specimens were collected in Argentina.

### Subserie Brasiliensis (Cabrera) Cabrera et S.E. Freire

*S. brasiliensis* (Spreng.) Less. var. *tripartitus* (DC.) Baker. **Buenos Aires**. Pdo. Escobar. Belén de Escobar, MGL 39 (SI), Pdo. Ensenada, Punta Lara, MGL & CCX 70, 71, 72 (SI)

*S. eruciformis* J. Rémy var. *Eruciformis*. **Mendoza**. Depto. Las Heras. Los Penitentes, AFW & MGL 1064, 1065 (SI),

*S. eruciformis* J. Rémy *brachycephallus* (Phil.) Cabrera. **Mendoza** Depto. Las Heras. Puente del Inca, AFW & MGL 1068, 1069 (SI)

*S. ganganensis* Cabrera. **Mendoza** Depto. Las Heras. Uspallata, AFW & MGL 1153 (SI)

*S. gilliesianus* Hieron. **Mendoza** Depto. Las Heras. Camino a Papagallos, AFW & MGL 1032 (SI). Frente al autódromo Gral. San Martín, AFW & MGL 1038 (SI). Estación Canota, MGL & AFW 129,130 (SI)

*S. glaber* Less. var. *glaber*. **Mendoza** Depto. San Rafael. Cerro Diamante, AFW & MGL 1207 (SI),

*S. goldsackii* Phil. **Mendoza** Depto. San Rafael entre Sonseado y Cañada Amarilla, AFW & MGL 1213 (SI). Depto. San Rafael Camino a Volcán Diamante, MGL & AFW 119 (SI). Depto. San Rafael. Camino a El Sosneado, AFW & MGL 1093 (SI)

*S. melanopotamicus* Cabrera. **Mendoza** Depto. Las Heras. Camino a Uspallata, polígono de tiro militar, AFW & MGL 1139, 1140, 1141(SI), Qda. de Santa Elena, AFW & MGL 1188 (SI). Camino a Mina Talcomín, AFW & MGL 1192, 1193 (SI)

*S. microphyllus* Phil. **Mendoza.** Depto. Las Heras. 32° 31′ 15″ S; 69° 02′ 4,8″ W; 2360 msm, AFW & MGL 1018 (SI). Caracoles de Villavicencio, AFW & MGL 1120 (SI)

*S. pampeanus* Cabrera. **San Luis.** Depto. Ayacucho. Quine, MGL55 y 56 (SI). **Córdoba.** Depto. San Alberto. Camino Altas Cumbres hacia Mina Clavero, AFW 908 (SI)

*S. pinnatus* Poir. var. *Pinnatus*. **Mendoza**. Depto. Luján. Cañada Grande, 8,5 km de Vallecito, AFW & MGL 1197 (SI). **Santiago del Estero.** Depto. Capital. Campus de la UCSE, MGL 148, 149, 150 (SI)

*S. ragonesei* Cabrera. **Mendoza**. Depto. Las Heras. Camino a Mina Talcomín, AFW & MGL 1189, 1190 (SI). Depto. San Rafael. Subiendo al Cerro Diamante, AFW & MGL 1208 (SI)

*S. riojanus* Cabrera var. *Riojanus*. **Mendoza** San Rafael. Puesto Vega del Burro, Ayo. Las Mangas, AFW & MGL 1211 (SI).

*S. rudbeckiifolius* Meyen et Walp. **Tucumán.** Depto. Tafí, AFW 916, 917, 918 (SI). C° Pelado, camino al C° de La Cruz, AFW 940, 942 (SI). **Salta.** Depto. Chicoana. Qda. Lapacheta, AMS & CCX 168 (SI). Depto. Cachi. 6 km de La Cuesta del Obispo, MGL, CCX & MNS 194 (SI)

*S. subulatus* D. Don. ex Hook. et Arn. var. *subulatus*. **Mendoza.** Depto. San Carlos. Ruta 40 camino a Agua del Toro, AFW & MGL 1200,1201 (SI). Depto. San Rafael. Puesto Vega del Burro, Ayo. Las Mangas, AFW & MGL 1209 (SI). Dique Agua de Toro, MGL & AFW 123 (SI). Depto. Malargüe. Malargüe, MGL & AFW 125 (SI). Depto. Las Heras. Camino a Uspallata desde Los Tambillos, MGL & AFW 142,145 (SI)

*S. subulatus* D. Don. ex Hook. et Arn. var. *erectus* Hook. et Arn. **Mendoza.** Depto. Luján. Camino a Potrerillos, AFW & MGL 1040, 1045, 1046 (SI). Depto. San Rafael. Dique Agua de Toro, MGL & AFW 122 (SI). Depto. Las Heras. Camino a Uspallata desde Los Tambillos, MGL & AFW 144 (SI). Depto. Luján de Cuyo. Camino a Potrerillos, MGL & AFW 146 (SI). **Salta** Depto. Cafayate. Alrededores de Cafayate, MGL, CCX & MNS 171 (SI)

*S. subulatus* D. Don. ex Hook. et Arn. var. *salsus* (Griseb.) Cabrera. **Salta**. Depto. Rosario de Lerma. Ruta 51 Km. 83 hacia Sta. Rosa de Tastil, MGL, CCX & MNS 213 (SI)

*S. uspallatensis* Hook. et Arn. **Mendoza**. Depto. Las Heras, 32° 31′ 15″ S – 69° 02′ 4.8″ W, AFW & MGL 1013 (SI). Los Hornillos, AFW & MGL 1024,1025 (SI). Puente del Inca, AFW & MGL 1070, 1071 (SI). Polvaredas, AFW & MGL 1078 (SI). Uspallata, Puesto Cuevas Norte, AFW & MGL 1171,1174 (SI). Caracoles de Villavicencio, MGL & AFW 132, 133, 134, 135, 137 (SI)

*S. viridis* Phil. var. v*iridis*. **Salta.** Depto. Los Andes. Base del Abra de Muñano 24° 21′ 51,8″ S; 66° 05′ 46,7″ W; 3750 msm, MGL,CCX & MNS 217 (SI)

*S. viridis* Phil. var. *radiatus* R. E. Fr. **Mendoza**. Depto. Las Heras. Qda. del Toro. 32° 28,3′ S; 69° 03,5′ W, AFW & MGL 1137 (SI). 16,6 km de Uspallata hacia el W. 32° 26,7′ S; 69° 13,9′ W; 2407 msm, AFW & MGL 1148 (SI). Uspallata, 13,2 km polígono de tiro militar, 32° 21,4′ S; 69° 12,7′ W; 2714 msm, AFW & MGL 1163, 1169 (SI). Depto. Las Heras. Pampa de Yalguaráz, 32° 10,2′ S; 69° 24,3′ W; 2249 msm, AFW & MGL 1181, 1177 (SI)

### Subserie Simplices (Cabrera) Cabrera et S. E. Freire

*S. angustissimus* Phil. **Neuquén**. Depto. Lácar. Cerro Chapelco, arriba del refugio Graeff, AMS *et al*. 95 (SI)

*S. grisebachii* Baker var. *grisebachii*. **Buenos Aires.** Pdo. Escobar. Camino a Paraná de Las Palmas, MGL 40 (SI)

*S. grisebachii* Baker var. *leptotus* Cabrera. **Bueno Aires.** Pdo. Merlo, MGL 1,3,6,8 (SI). Pdo. Exaltación de La Cruz. Los Cardales, MGL & AFW 87 (SI)

*S. grisebachii* Baker var. *schizotus* Cabrera. **Bueno Aires** Bs. As. Pdo. Merlo, MGL 10, 34 (SI). Pdo. Escobar. El Cazador, MGL 42 (SI)

*S. hieronymi* Griseb. **Tucumán**. Depto. Tafí. San Javier, entrada del Cristo, AFW 926, 927 (SI). **Salta.** Depto. Chicoana; 3348 msm, AMS & CCX 178 (SI). Depto. Rosario de Lerma. Costado de ruta 51 Km. 16,5, MGL, CCX & MNS 178 (SI). Depto. Cachi. Ruta 33 Km 66. Parque Nac. Los Cardones, MGL, CCX & MNS 200 (SI)

*S. linariifolius* Poepp.ex DC. var. *subtomentosus* Cabrera. **Mendoza**. Depto. Las Heras. Cuesta de Bonilla, 32° 39,5′ S; 69° 11,05′ W; 3087 msm, AFW & MGL 1183 (SI)

*S. octolepis* Griseb. var. *saltensis* (Hicken) Cabrera et Zardini. **Tucuman** Depto. Tafí. Atrás de Loma Pelada, AFW 915 (SI). **Salta** Depto. Chicoana. Qda. Lapacheta, AMS & CCX 1692 (SI)

### Subserie Viscosi (Baker) Cabrera et S. E. Freire

*S. crepidifolius* DC. **Salta.** Depto. Santa Victoria. Abra de Lizoite. Ruta 7 Km 54; 4500 msm, AMS & CCX 222(SI)

## Authors’ original submitted files for images

Below are the links to the authors’ original submitted files for images. Authors’ original file for figure 1 Authors’ original file for figure 2
