# Supplementary figures and images for: Deep cytogenetics analysis reveals meiotic recombination depletion in species of Senecio (Asteraceae)
Source: Bot Stud. 2013 Aug 27;54:20. doi: 10.1186/1999-3110-54-20 (PMC5432766; doi:10.1186/1999-3110-54-20)

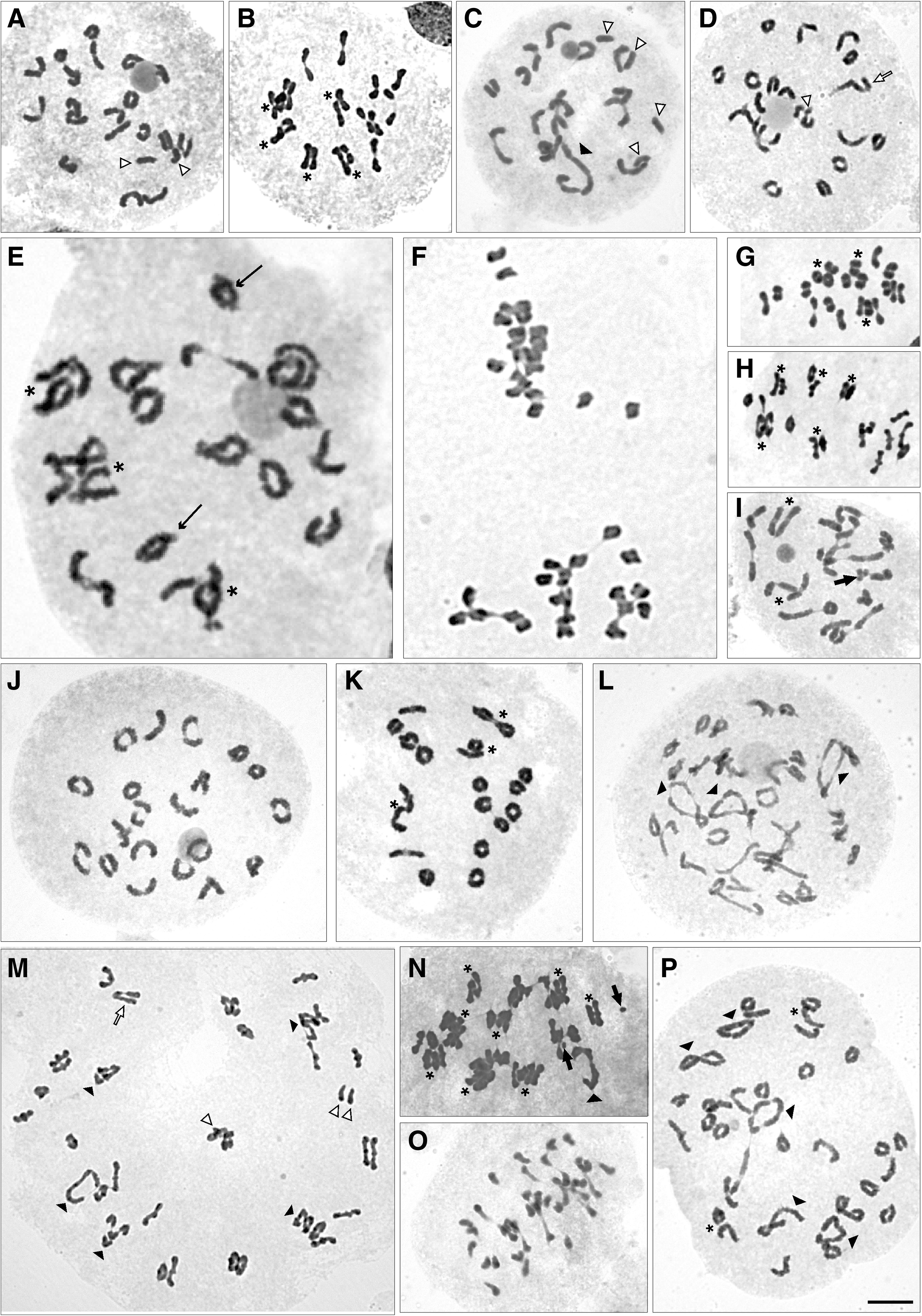

Supplement: Supplementary file 1 — Authors’ original file for figure 1 [file 40529_2011_19_MOESM1_ESM.tif]

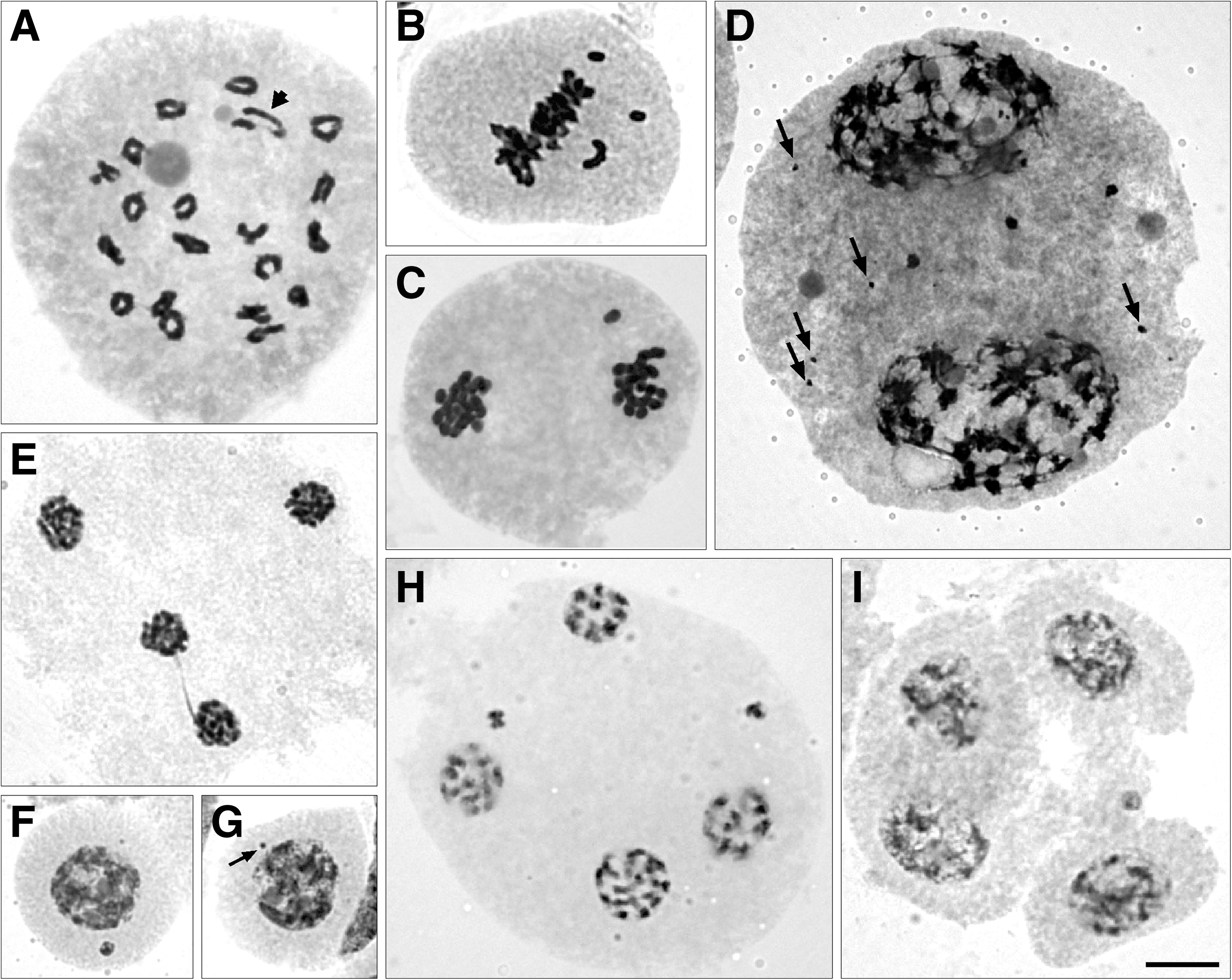

Supplement: Supplementary file 2 — Authors’ original file for figure 2 [file 40529_2011_19_MOESM2_ESM.tif]
